# Parent–Adolescent Communication, Siblings and Adolescent Coping Strategies

**DOI:** 10.3390/bs16071163

**Published:** 2026-07-10

**Authors:** Megha Garg, Mellissa S. Gordon, Christine M. Ohannessian

**Affiliations:** 1 Department of Human Development and Family Sciences, University of Delaware, 111 Alison Hall West, Newark, DE 19716, USA; meghagar@udel.edu; 2 Department of Human Development and Family Science, Florida State University, SAN, Sandels, 225, 120 Convocation Way, Tallahassee, FL 32304, USA; christine.ohannessian@gmail.com

**Keywords:** coping strategies, adolescents, birth order, gender dyad, parent–adolescent communication

## Abstract

Relationships between adolescents and their siblings may be associated with birth order and gender. Additionally, the spillover hypothesis suggests that emotions and behaviors in one family subsystem can affect others. Accordingly, the quality of parent–child relationship might “spill over” into the sibling dynamic, thereby influencing adolescent development. This study examines whether birth order and sibling gender dyads are significantly associated with the adolescents’ use of active, distraction, and problem-focused coping strategies. Additionally, it tests whether gender dyads moderate this association. Data came from a large-scale longitudinal dataset of adolescent participants in the Northeast region of the United States, *N* = 1428 (*M_age_* = 12.75, *SD* = 0.71, at Time 1). Direct and moderating effects are tested using regression-based structural equation analyses. Results suggest that birth order was significantly associated with the adolescents’ use of coping strategies when the adolescent was older and within a mixed-gender dyad. Additionally, significant interactions are found for same and mixed-gender dyads when the adolescent was older. Findings provide foundational support from which to explore the role of birth order and sibling gender dyads in adolescent development. Furthermore, it provides a basis for mental health practitioners and counselors working with adolescents to leverage the sibling relationship in promoting healthy coping strategies.

## 1. Introduction

Sibling relationships play a significant role in the lives of children and adolescents (Blyth et al., 1982). The quality of these relationships has been linked to adolescents’ well-being, self-esteem, and social development (for instance, Kim et al., 2007; Yeh & Lempers, 2004). Furthermore, sibling support is a significant aspect of the sibling relationship quality and has been found to be negatively associated with externalizing problems among adolescents (Branje et al., 2004).

In addition to sibling support, other factors that have been found to be associated with adolescent outcomes, such as gender and birth order (Arora et al., 2024). During adolescence, same-gender siblings were found to have a closer relationship as compared to mixed-gender dyads (Barroso, 2011). Regarding birth order, having an older sibling (as opposed to a younger sibling) was identified as a protective factor for adolescents against externalizing problems (Fry et al., 2021). Given the salience of both gender and birth order in development, it is quite possible that the two may interact with each other to influence adolescents’ development.

Furthermore, according to Liga et al. (2020), adolescence is a transitional phase in which individuals experience several psychological and social changes. These changes place additional demands on the adolescents to develop new behavioral responses (Liga et al., 2015). These responses, however, are not consistent among individuals within this age group, as it has been observed that some adolescents can effectively negotiate these challenges, while others may find it more challenging (Markova & Nikitskaya, 2017). Accordingly, one of the important attributes that may contribute to adolescents’ psychosocial adjustment is their ability to cope with stress (Hashmi, 2013).

Particularly, coping strategies during adolescence have been found to be associated with later adult outcomes, such as adult convictions (Aebi et al., 2014), and drug abuse and dependency among incarcerated youth (Eftekhari et al., 2004). Therefore, given the association between adolescent coping strategies and adult outcomes, addressing the factors that influence the development of coping strategies is salient to design intervention strategies.

### 1.1. Family Systems Theory

Family systems theory proposes that families consist of subsystems that are interrelated and influence each other mutually (Whitchurch & Constantine, 1993). This interrelatedness between the subsystems is explained by the congruence (spillover) and compensation hypotheses. According to the spillover hypothesis, mood, affect, or behavior, directly transfers from one setting to another (Repetti, 1987). This process involves the expression of feelings that occurred in one system (for example, parent–child dyad) into another (for example, sibling dyad; Erel & Burman, 1995). The direction of this expression can be positive or negative. A positive expression is such that more positive (or negative) parent–child relationships are associated with more positive (or negative) sibling relationships (Derkman et al., 2011). Conversely, according to the compensation hypothesis, negative relationships with some family members might be compensated through more positive relationships with other family members (Derkman et al., 2011). Particularly, research suggests that there is a positive association between parent–child and sibling relationships (e.g., McHale et al., 2006; Noller, 2005). Accordingly, the quality of the parent–child relationship might “spill over” into the sibling dynamic, thereby influencing adolescent development.

Furthermore, according to family systems theory, communication within a family system defines its characteristics (Smith & Hamon, 2022). This communication can be verbal, through the message’s content, or non-verbal, such as gestures or postures (Smith & Hamon, 2022), and shapes the behavior of the family members. Therefore, given the salience of communication patterns within and across family subsystems, the present study aims to study the communication between parents and adolescents and their association with adolescents’ coping strategies.

### 1.2. Literature Review

#### 1.2.1. Coping Strategies

According to Lazarus and Folkman (1984), coping consists of cognitive and behavioral efforts that are constantly changing to manage internal and external demands that exceed a person’s resources. These strategies undergo significant changes during the transition from late childhood to early adolescence (Zimmer-Gembeck & Skinner, 2016) and become more diverse. With these changes, classifying coping strategies into various categories has become increasingly challenging. For example, Connor-Smith et al. (2000) argue that coping strategies should be classified into two categories: approach or avoidant. However, using such a dichotomous approach might result in several strategies being stranded that might not fall into these categories. On the contrary, using several categories, such as the eight suggested by Malooly et al. (2017), might result in overcomplication and overlap in conceptualizing the categories of coping strategies. Studies such as that by Ohannessian (2012) propose a more manageable approach of using three categories of coping strategies: active, distraction, and problem-focused.

#### 1.2.2. Active Coping

Active coping refers to the process of making deliberate efforts to either remove or cope with a stressful situation (Carver et al., 1989). Studies have demonstrated an association between the use of active coping strategies and positive mental health. For example, Frydenberg and Lewis (2009) found that using active coping strategies positively predicted well-being among adolescents, using an Australian sample. Similarly, Seiffge-Krenke et al. (2009) found a negative association between adolescents’ use of active coping strategies and stress.

#### 1.2.3. Distraction Coping

According to Carver et al. (1989), distraction coping refers to the process of mental disengagement through activities aimed at distracting a person from thinking about a problem. Studies have found that this can be an adaptive strategy to channel attention towards wellness behaviors, such as speaking to a friend or taking a walk (Baum et al., 2009; Marsac et al., 2013; Prinstein et al., 1996). Particularly among female adolescents, distraction coping was found to be associated with reduced trauma-related symptoms (Elzy et al., 2013).

#### 1.2.4. Problem-Focused Coping

Problem-focused coping strategies include confronting the event or the situation that is causing distress (Lazarus & Folkman, 1984). Cong et al. (2021) found a negative association between problem-focused coping strategy and depression among adolescents. Similarly, Zammuner (2019) found that problem-solving coping strategy was associated with lower levels of social loneliness and higher levels of life satisfaction.

### 1.3. Parent–Adolescent Communication and Coping Strategies

Evidence supports the crucial role of parental relationships in the development of adolescent coping (e.g., Stroud & Fitts, 2017). In particular, parent–adolescent communication is a significant indicator of parent–adolescent relationship quality (Ohannessian, 2013) and is significantly associated with the development of coping strategies during adolescence (Simpson et al., 2020).

Furthermore, this association was found to be associated with the gender of the parent and adolescent. For example, Simpson et al. (2020) found that adolescent–mother communication positively predicted emotion-focused coping strategies among boys. On the contrary, adolescent–father communication negatively predicted emotion-focused coping strategies among boys. Additionally, the authors also found that mother–adolescent communication positively predicted the use of active coping among girls. However, father–adolescent communication did not significantly predict any coping strategies among girls. Therefore, since the influence of parental communication on adolescent coping varies based on the parents’ gender, it is crucial to examine them separately to get a detailed understanding of the association between parent–adolescent communication and adolescent coping strategies.

Despite the aforementioned findings, the literature examining parent–adolescent communication and coping strategies has yielded inconsistent results. On the one hand, some studies have reported that parent–adolescent communication does not predict adolescents’ use of coping strategies (e.g., Suh & Shin, 2016). On the other hand, some studies have found a significant positive relation between parent–adolescent communication and adolescents’ use of coping strategies (e.g., Simpson et al., 2020). As such, additional studies are needed to further disentangle this association.

### 1.4. Role of Siblings

Research indicates that siblings typically spend about 50 percent of their time together during early adolescence. Furthermore, a significant majority of children are raised in households with one or more siblings, highlighting the importance of sibling relationships in developmental contexts (Dunifon et al., 2017). In fact, the longest-lasting social relationships are typically those with siblings (McHale et al., 2012). However, these relationships are often influenced by demographic and structural factors such as gender and birth order. Same-gender siblings have been found to have closer relationships than mixed-gender siblings during adolescence (Barroso, 2011). Within these dyads, sisters reported the closest relationships (Riggio, 2006; Scharf et al., 2005). Regarding its influence on adolescent development, Doughty et al. (2015) found that adolescents in mixed-gender sibling dyads reported higher romantic intimacy as compared to those in same-gender dyad.

Furthermore, birth order and age-related differences within sibling relationships can create an inherent hierarchical dynamic that might influence the effects of support and warmth provided within sibling dyads (Feinberg et al., 2012). Younger siblings are more inclined to seek guidance or support from their elder siblings because they perceive them as strong and caring (Fry et al., 2021). Nonetheless, younger siblings actively participate in the sibling subsystem, exchanging support and thereby influencing the sibling relationship in both directions (Cox & Paley, 1997). Lastly, older-born siblings are less likely to engage in risky behaviors as compared to younger siblings (Argys et al., 2006).

### 1.5. Additional Factors of Interest

The type of coping strategy used might also be influenced by factors such as the quality of the sibling relationship, mother–adolescent and father–adolescent communication, the adolescents’ age, and race. For example, in a study by Garg et al. (2025), the authors found conflicting results of the association between sibling relationship quality and adolescent coping based on time. Particularly, the authors found that sibling support at an earlier time predicted adolescents’ use of active, distraction, and problem-focused coping strategies negatively. However, support from siblings at a later time predicted the three coping strategies positively. Additionally, age was also found to influence adolescents’ use of coping strategies. More specifically, Vierhaus and Lohaus (2009) found that the use of problem-solving coping increased with age, particularly during childhood. Furthermore, the use of distraction coping also increased as the age progressed (Eschenbeck et al., 2018). The authors also concluded that during early adolescence, there is a shift towards more active coping strategies (Eschenbeck et al., 2018).

According to Estlein (2021), parent–adolescent communication is the interpersonal interaction between the parent and the adolescent and contains various degrees of responsiveness and control. On one hand, where responsiveness is the degree of synchronization and parental support, control refers to the use of rigid, confrontational, and stern communication patterns within the dyad (Estlein, 2021). Particularly during adolescence, this communication is constantly renegotiated, and the process is often followed by conflict (Keijsers & Poulin, 2013), making it more challenging.

Quality communication between parents and adolescents is associated with healthy youth development. For example, Cui et al. (2021) found that better parent–adolescent communication was associated with higher well-being among U.S. female young adults. A negative correlation was identified between effective parent–adolescent communication and externalizing problems in adolescents (Davidson & Cardemil, 2009). Additionally, poor communication between parents and adolescents was linked to increased aggression in school settings (Lambert & Cashwell, 2004) and a higher likelihood of engaging in delinquent behaviors (Clark & Shields, 1997).

Literature suggests that parent–adolescent communication differs based on the parent’s gender. Mothers are typically more inclined to communicate more actively, frequently, and emotionally with children (Barnes & Olson, 1985; Shek, 2000) as compared to fathers. Additionally, mothers may receive more information about the children’s problems and, therefore, be able to provide timely support (Darling et al., 2006; Pantaleao & Ohannessian, 2019). Conversely, fathers often provide increased problem-solving strategies and autonomy support to adolescents (Lamb & Lewis, 2013; Huang et al., 2021). Furthermore, studies have found that both mothers’ and fathers’ communication significantly predicted depressive symptoms (Zhang et al., 2021) and externalizing symptoms (Majid et al., 2023) among adolescents.

Race and ethnicity may also influence the type of coping strategies adolescents use. Chapman and Mullis (2000) compared African American and Caucasian adolescents on their use of coping strategies. The authors found that adolescents identifying themselves as African American reported more frequent use of distraction, self-reliance, spiritual, and social support as compared to their Caucasian counterparts. Additionally, Caucasian adolescents reported using venting and avoidance more frequently than African Americans.

### 1.6. Significance of the Study

Based on the findings from the literature and the premises of the family systems theory, the present study aims to extend the literature regarding coping strategies and the dynamics of sibling relationships. First, using a longitudinal approach, this study will examine whether birth order and gender dyads (whether they belong to same or mixed-gender dyads) of siblings are directly associated with the adolescents’ use of active, distraction, and problem-focused coping strategies). Furthermore, this study will also examine whether gender dyad moderates the association between birth order and adolescents’ use of the three coping strategies.

The following hypotheses are explored:

**H1.**  *Birth order will significantly predict adolescents’ use of three coping strategies (active, distraction, and problem-focused), such that older siblings would have higher reports of the three coping strategies as compared to younger siblings.*

**H2.**  *Sibling gender dyad will significantly predict adolescents’ use of three coping strategies (active, distraction, and problem-focused) such that siblings in the same-gender dyad will have higher use of the three coping strategies as compared to siblings in a mixed-gender dyad.*

**H3.**  *The association between birth order and adolescents’ use of three coping strategies (active, distraction, and problem-focused) is moderated by gender dyad.*

The present study extends the literature in several ways. First, even though previous studies have examined the associations between birth order and gender dyads and adolescent development (e.g., Argys et al., 2006; Doughty et al., 2015) none has examined their association with coping strategies. Given the association between adolescent coping strategies and adult outcomes, it is important to examine coping strategies in this population and provide interventions, if required. Furthermore, the present study isolates the unique effects of mothers and fathers communication by first, testing the hypotheses by including mothers’ communication as a covariate; and second testing the hypotheses by including fathers’ communication as a covariate, thus examining the spillover hypothesis. Since the communication styles of parents differ based on their gender (for example, Huang et al., 2021 and Shek, 2000), it is crucial to examine their unique effects on adolescent coping. Next, given the salience of siblings in individual development, it is crucial to examine whether the structure of this relationship, i.e., their birth order and gender composition influence the development of the three coping strategies. Even though the previous studies have established the importance of birth order and gender dyad in adolescent development (for example, Arora et al., 2024), none have done so for adolescent coping. Particularly, no study has examined whether there is an interaction between the adolescent sibling birth order and their gender dyad and whether it is associated with adolescents’ active, distraction and problem-focused coping strategies. The present study addresses this gap by examining whether the association between birth order and adolescents’ use of three coping strategies (active, distraction, and problem-focused) is moderated by gender dyad. This assumption is in line with the previous literature, which has found a significant interaction between these two variables in influencing adolescent development (e.g., Adamov et al., 2024). Furthermore, given the significance of sibling relationship quality in the development of adolescent coping strategies, the present study includes sibling relationship quality from Time 1 and Time 2 as a covariate. Lastly, acknowledging the potential influence of parent–adolescent communication (separately for mothers and fathers), age and race, this study includes these variables as covariates.

## 2. Materials and Methods

### 2.1. Sample and Procedure

The present study utilized a sample from a larger longitudinal study exploring factors that protected adolescents from developing psychological problems. The researchers contacted the parents of students by mailing a letter describing the study. In the letter, the parents were requested to contact the research team by email, mail or phone if they did not want their student to participate in the study. Out of the total parents contacted, only 2% refused participation. Data collection was completed across three time points: fall of 2016 (T1), spring of 2017 (T2), and spring of 2018 (T3). Inclusion criteria were ability to understand the survey in English and no report of severe developmental disorder. Students who provided assent were asked to complete self-report surveys administered by trained research staff. The majority of the adolescents, 94%, agreed to participate and signed the assent form.

The present study includes data from all three waves, and the final sample consisted of *N* = 1428 adolescents. The mean age of participants at T1 was *M_age_* = 12.75 years (*SD* = 0.71) and ranged from 11 to 15 years. Approximately half of the participants self-identified as males and half as females. Among the participants, 52.15% self-identified themselves as White, 8.75% as Black or African American, 19.88% as Hispanic or Latino, and 19.22% as belonging to several other races/ethnicities. Within the sample, 57.14% of participants were younger than their siblings and 51.80% reported that they and their siblings were of the same-gender (same-gender dyad). Additional details regarding sociodemographic characteristics of the participants are given in Table 1.

### 2.2. Measures

#### 2.2.1. Coping Strategies

The 52-item Children’s Coping Strategies Checklist (CCSC; Program for Prevention Research, 1999) was used to measure active, distraction, and problem-focused coping strategies. Data for the outcomes were taken from Time 3. Example items for the three coping strategies included active coping: “*You did something to solve the problem*”; distraction coping: “*You listened to music*”; and problem-focused coping: “*You did something to make things better*”. Responses ranged from *1 = never* to *4 = most of the time*. The scale showed adequate reliability and validity (Ayers et al., 1996).

#### 2.2.2. Birth Order

At Time 2, the ages of both the participant and the sibling closest to them in age (assigned as the focal sibling) were recorded. Two indicator terms were then created to indicate their age differences: participant older: *1 = participant’s age at Time 2 was greater than sibling’s age at Time 2*, *0 = participant’s age at Time 2 is less than the sibling’s age at Time 2* and participant younger: *1 = participant’s age at Time 2 is less than the sibling’s age at Time 2*, *0 = participant’s age at Time 2 is greater than the sibling’s age at Time 2*.

#### 2.2.3. Gender Dyad

The gender of both the participant and their sibling was used to create a variable that indicated whether they were in the same-gender dyad or a mixed-gender dyad. The indicator terms were created such that: same-gender dyad: *1 = gender of participant is same as the sibling’s gender*, *0 = gender of participant is not same as the sibling’s gender*; mixed-gender dyad: *1 = gender of participant is not same as the sibling’s gender*, *0 = gender of participant is same as the sibling’s gender*.

#### 2.2.4. Covariates

##### Parent–Adolescent Communication

Quality of parent–adolescent communication was assessed using the Parent–Adolescent Communication Scale (PACS; Barnes & Olson, 2003). This scale consists of 20 items and two subscales: open communication (e.g., “*If I were in trouble*, *I could tell my mother/father*”) and communication problems (e.g., “*When we are having problem*, *I often give my mother/father the silent treatment.*”). Each item was asked twice, once for each parent and responses were recorded on a 5-point Likert scale, ranging from *1 = strongly disagree* to *5 = strongly agree*. In the current study, communication was assessed separately for each parent from Time 1. Furthermore, the Cronbach’s alpha was 0.92 for mother–adolescent communication and 0.94 for father–adolescent communication.

##### Sibling Relationship Quality

Quality of sibling relationships was measured at Time 1 and Time 2 using the Sibling Relationship Questionnaire (SRQ; Slomkowski et al., 2001). Adolescents reported their perception of warmth between them and their target sibling on a 7-point Likert scale ranging from *1 = never* to *7 = always*. Sample item included “*Lets you know he/she really cares about you.*”. The Cronbach’s alpha for this scale was 0.89 (Finan et al., 2018).

##### Race

Race was included from Time 1. Participants were asked: *Do you think of yourself as…?* Responses were recorded as *0 = White*, *1 = Black or African American*, *2 = Hispanic or Latino*, and *3 = Other*. Race was dummy-coded with White as the reference category and each dummy variable was included in the analysis.

##### Age

At Time 1, participants were asked to report their age (in years).

### 2.3. Data Analyses

Data analyses were completed using the statistical software package STATA 17. First, the direct effect of birth order and sibling gender dyad (whether the participant and the sibling belonged to the same-gender dyad or different) on the three coping strategies: active, distraction and problem-focused was tested. This was done separately for mothers’ and fathers’ communication using the SEM function in STATA 17. Next, to test the moderating effect of gender dyad, four interaction terms were created by multiplying birth order by gender dyad (e.g., participant was older X same-gender dyad’). Next, again using the SEM function in STATA, each of the four interaction terms were included in models, separate for mothers’ and fathers’ communication and including the participant’s age and race at Time 1, and sibling relationship quality at Time 1 and Time 2 as covariates. Missing data were handled using the Full Information Maximum Likelihood (FIML).

## 3. Results

### 3.1. Descriptive Statistics

Table 1 provides the descriptive statistics of the study variables. According to the findings, adolescents reported higher use of active (*M* = 2.35, *SD* = 0.77) and problem-focused (*M* = 2.35, *SD* = 0.77) coping strategies as compared to distraction coping strategies (*M* = 2.19, *SD* = 0.61). Furthermore, the quality of sibling relationship declined from Time 1 (*M* = 4.82, *SD* = 1.55) to Time 2 (*M* = 4.74, *SD* = 1.60). Adolescents also reported better quality of communication with mothers (*M* = 71.08, *SD* = 14.50) as compared to fathers (*M* = 70.18, *SD* = 15.18).

### 3.2. Hypotheses Testing

#### 3.2.1. When the Participant Was Older

**Same-gender dyad.** According to Table 2, Table 3, Table 4, Table 5, Table 6 and Table 7, when the participant was older than their sibling and were in the same-gender dyad, mother–adolescent communication was positively associated with adolescents’ use of all three coping strategies: active (*b* = 0.01, *p* < 0.05), distraction (*b* = 0.01, *p* < 0.05), and problem-focused (*b* = 0.01, *p* < 0.05). Similarly, father–adolescent communication was significantly and positively associated with the adolescents’ use of active (*b* = 0.02, *p* < 0.05), distraction (*b* = 0.01, *p* < 0.05), and problem-focused (*b* = 0.02, *p* < 0.05) coping strategies.

According to Table 2, Table 3 and Table 4, testing of direct effects revealed that in the context of mother–adolescent communication, being older than their sibling was not significantly associated with the use of active (*b* = −0.02, *p* > 0.05), distraction (*b* = 0.05, *p* > 0.05), and problem-focused (*b* = −0.02, *p* > 0.05) coping strategies. Furthermore, direct testing for gender dyads suggested that being in the same-gender dyad was significantly and negatively associated with the adolescents’ use of active (*b* = −0.23, *p* < 0.05) and problem-focused (*b* = −0.21, *p* < 0.05) coping strategies but not distraction (*b* = −0.09, *p* > 0.05).

Regarding covariates (Table 2, Table 3 and Table 4), it was found that in the context of mother–adolescent communication, sibling relationship quality at Time 1 was not significantly associated with adolescents’ use of active coping strategy (*b* = −0.05, *p* > 0.05), but was significantly and negatively associated with distraction (*b* = −0.06, *p* < 0.05), and problem-focused (*b* = −0.06, *p* < 0.05) coping strategies. Conversely, sibling relationship quality at Time 2 was significantly and positively associated with adolescents’ use of active (*b* = 0.17, *p* < 0.05), distraction (*b* = 0.11, *p* < 0.05), and problem-focused (*b* = 0.16, *p* < 0.05) coping strategies.

Additionally, identifying as Black or African American race was not significantly associated with the adolescents’ use of active (*b* = −0.04, *p* > 0.05), distraction (*b* = 0.03, *p* > 0.05), and problem-focused (*b* = −0.04, *p* > 0.05) coping strategies. However, identifying as Hispanic or Latino was significantly and negatively associated with their active (*b* = −0.03, *p* < 0.05) and problem-solving (*b* = −0.29, *p* < 0.05) coping but not with distraction (*b* = −0.14, *p* > 0.05). Furthermore, identifying as belonging to other racial categories was also not significantly associated with the adolescent’s use of active (*b* = −0.04, *p* > 0.05), distraction (*b* = −0.08, *p* > 0.05), and problem-focused (*b* = −0.03, *p* > 0.05) coping strategies. Similarly, the age of the adolescents at Time 1 was not significantly associated with their use of active (*b* = 0.04, *p* > 0.05), distraction (*b* = 0.00, *p* > 0.05), and problem-focused (*b* = 0.04, *p* > 0.05) coping strategies (Table 2, Table 3 and Table 4).

Next, based on moderation analyses (Table 2, Table 3 and Table 4), in the context of mother–adolescent communication, the interaction between same-gender dyad and when the participant was older was significant for both active (*b* = 0.29, *p* < 0.05) and problem-focused coping strategies (*b* = 0.31, *p* < 0.05) but not for distraction (*b* = 0.17, *p* > 0.05).

Regarding father–adolescent communication (Table 5, Table 6 and Table 7), being older than the sibling was not significantly associated with higher use of active (*b* = 0.05, *p* > 0.05), distraction (*b* = 0.09, *p* > 0.05), and problem-focused (*b* = 0.04, *p* > 0.05) coping strategies. Additionally, according to the findings of the direct effects of gender dyads, being in the same-gender dyad was significantly and negatively associated with the adolescents’ use of active (*b* = −0.19, *p* < 0.05) and problem-focused (*b* = −0.16, *p* < 0.05) coping strategies but not distraction (*b* = −0.05, *p* > 0.05).

Regarding covariates (Table 5, Table 6 and Table 7), sibling relationship quality at Time 1 was marginally associated with adolescents’ use of distraction coping strategies (*b* = −0.04, *p* = 0.049). However, it was not significantly associated with adolescents’ use of active coping (*b* = −0.02, *p* > 0.05) or problem-focused coping strategies (*b* = −0.04, *p* > 0.05). On the contrary, sibling relationship quality at Time 2 was significantly and positively associated with adolescents’ use of all three coping strategies: active (*b* = 0.14, *p* < 0.05), distraction (*b* = 0.09, *p* < 0.05), and problem-focused (*b* = 0.13, *p* < 0.05).

Furthermore, identifying as Black or African American was not significantly associated with adolescents’ use of any of the three coping strategies: active (*b* = −0.00, *p* > 0.05), distraction (*b* = 0.05, *p* > 0.05), and problem-focused (*b* = 0.00, *p* > 0.05). However, identifying as Hispanic or Latino was significantly and negatively associated with adolescent’s use of active (*b* = −0.20, *p* < 0.05), distraction (*b* = −0.07, *p* < 0.05), and problem-focused (*b* = −0.19, *p* < 0.05) coping strategies. Furthermore, identifying as belonging to other racial category was not significantly associated with any of the three coping strategies: active (*b* = 0.01, *p* > 0.05), distraction (*b* = −0.05, *p* > 0.05), and problem-focused (*b* = 0.02, *p* > 0.05). Similarly, the age of the adolescents was not significantly associated with their use of active (*b* = 0.02, *p* > 0.05), distraction (*b* = −0.02, *p* > 0.05), or problem-focused (*b* = 0.02, *p* > 0.05) coping strategies (Table 5, Table 6 and Table 7).

Furthermore, in the context of father–adolescent communication (Table 5, Table 6 and Table 7), the interaction between same-gender dyad and when the participant was older was significant for only active (*b* = 0.25, *p* < 0.05) and problem-focused coping strategies (*b* = 0.25, *p* < 0.05) but not for distraction (*b* = 0.13, *p* > 0.05).

**Mixed-gender dyad.** According to Table 8, Table 9, Table 10, Table 11, Table 12 and Table 13, for mixed-gender dyads and when the participant was older, mother–adolescent communication was significantly and positively associated with the adolescents’ use of active (*b* = 0.01, *p* < 0.05), distraction (*b* = 0.01, *p* < 0.05), and problem-focused (*b* = 0.01, *p* < 0.05) coping strategies. Similarly, father–adolescent communication was significantly and positively associated with adolescents’ use of active (*b* = 0.02, *p* < 0.05), distraction (*b* = 0.01, *p* < 0.05), and problem-focused (*b* = 0.02, *p* < 0.05) coping strategies.

For the mixed-gender dyad (Table 8, Table 9 and Table 10), testing for direct effects in the context of mother–adolescent communication revealed that being older than the sibling was significantly and positively associated with the adolescents’ use of active (*b* = 0.27, *p* < 0.05), distraction (*b* = 0.22, *p* < 0.05), and problem-focused (*b* = 0.28, *p* < 0.05) coping strategies. Additionally, when testing gender dyads, findings revealed that being in the mixed-gender dyad was significantly and positively associated with active (*b* = 0.23, *p* < 0.05) and problem-focused coping (*b* = 0.21, *p* < 0.05) but not distraction coping (*b* = 0.08, *p* > 0.05).

Testing of covariates (Table 8, Table 9 and Table 10) revealed that sibling relationship quality at Time 1 was significantly and negatively associated with adolescents’ use of all), distraction (*b* = −0.06, *p* < 0.05), and problem-focused (*b* = −0.06, *p* < 0.05) coping strategies, but not with active (*b* = −0.05, *p* > 0.05) coping strategy. However, sibling relationship quality at Time 2 was significantly and positively associated with adolescents’ use of active (*b* = 0.17, *p* < 0.05), distraction (*b* = 0.11, *p* < 0.05), and problem-focused (*b* = 0.16, *p* < 0.05) coping strategies.

Regarding race and age (Table 8, Table 9 and Table 10), identifying as Black or African American was not significantly associated with adolescents’ use of any of the three coping strategies: active (*b* = −0.04, *p* > 0.05), distraction (*b* = 0.03, *p* > 0.05), and problem-focused (*b* = −0.04, *p* > 0.05). However, identifying as Hispanic or Latino was significantly and negatively associated with the adolescent’s use of active (*b* = −0.30, *p* < 0.05) and problem-focused (*b* = −0.29, *p* < 0.05) coping strategies, but not with distraction (*b* = −0.14, *p* > 0.05). Furthermore, identifying as belonging to other racial category was not significantly associated with the adolescent’s use of any of the three coping strategies: active (*b* = −0.04, *p* > 0.05), distraction (*b* = −0.08, *p* > 0.05), and problem-focused (*b* = −0.03, *p* > 0.05). Similarly, participant’s age at Time 1 was not significantly associated with the adolescents’ use of active (*b* = 0.04, *p* > 0.05), distraction (*b* = −0.00, *p* > 0.05), and problem-focused (*b* = 0.04, *p* > 0.05) coping strategies.

In the context of mother–adolescent communication, the results of moderation analysis (Table 8, Table 9 and Table 10) revealed that the interaction between mixed-gender dyad and when the participant was older was significant for active (*b* = −0.29, *p* < 0.05), and problem-focused (*b* = −0.31, *p* < 0.05) coping but not for distraction (*b* = −0.17, *p* > 0.05) coping strategies.

In the context of fathers’ communication (Table 11, Table 12 and Table 13), it was found that being older than the sibling was significantly and positively associated with the adolescents’ use of all three coping strategies: active (*b* = 0.29, *p* < 0.05), distraction (*b* = 0.23, *p* < 0.05), and problem-focused (*b* = 0.30, *p* < 0.05). Furthermore, it was also found that being in a mixed-gender dyad was significantly and positively associated with only active (*b* = 0.19, *p* < 0.05) and problem-focused coping (*b* = 0.16, *p* < 0.05), but not distraction (*b* = 0.05, *p* > 0.05).

Testing for covariates (Table 11, Table 12 and Table 13) revealed that sibling relationship quality at Time 1 was only negatively associated with adolescents’ use of distraction coping (*b* = −0.04, *p* < 0.05). The association between sibling relationship quality at Time 1 and active (*b* = −0.02, *p* > 0.05) and problem-focused coping (*b* = −0.04, *p* > 0.05) was not significant. Conversely, sibling relationship quality at Time 2 was significantly and positively associated with adolescents’ use of active (*b* = 0.14, *p* < 0.05), distraction (*b* = 0.09, *p* < 0.05), and problem-focused (*b* = 0.13, *p* < 0.05) coping strategies.

Furthermore, according to Table 11, Table 12 and Table 13, identifying as Black or African American was not associated with adolescents’ use of active (*b* = −0.00, *p* > 0.05), distraction (*b* = 0.05, *p* > 0.05), and problem-focused (*b* = 0.00, *p* > 0.05) coping strategies. However, identifying as Hispanic or Latino was significantly and negatively associated with adolescent’s use of active (*b* = −0.20, *p* < 0.05) and problem-focused (*b* = −0.19, *p* < 0.05) coping strategies, but not with distraction (*b* = −0.07, *p* > 0.05) coping. Additionally, identifying as belonging to other racial groups was not associated with the adolescent’s use of any of the three coping strategies: active (*b* = 0.01, *p* > 0.05), distraction (*b* = −0.05, *p* > 0.05), and problem-focused (*b* = 0.02, *p* < 0.05). Similarly, age at Time 1 was not associated with the adolescents’ use of any of the three coping strategies: active (*b* = 0.02, *p* > 0.05), distraction (*b* = −0.02, *p* > 0.05), and problem-focused (*b* = 0.02, *p* > 0.05).

Furthermore, in the context of father–adolescent communication, the interaction between being in a mixed-gender dyad and when the participant was older (Table 11, Table 12 and Table 13) was significant only for active (*b* = −0.25, *p* < 0.05) and problem-focused (*b* = −0.25, *p* < 0.05) coping but not for distraction (*b* = −0.13, *p* > 0.05) coping strategies.

#### 3.2.2. When the Participant Was Younger

**Same-gender dyad.** Within the same-gender dyad, when the participant was younger, according to Table 14, Table 15, Table 16, Table 17, Table 18 and Table 19, mother–adolescent communication was significantly and positively associated with the adolescents’ use of active (*b* = 0.01, *p* < 0.05), distraction (*b* = 0.01, *p* < 0.05), and problem-focused (*b* = 0.01, *p* < 0.05) coping strategies. Similarly, father–adolescent communication was significantly and positively associated with the adolescents’ use of the three coping strategies: active (*b* = 0.02, *p* < 0.05), distraction (*b* = 0.01, *p* < 0.05), and problem-focused (*b* = 0.02, *p* < 0.05).

According to the results of direct effects, in the context of mother–adolescent communication (Table 14, Table 15 and Table 16), it was found that being younger than the sibling was not significantly associated with their use of active (*b* = −0.02, *p* > 0.05), distraction (*b* = −0.06, *p* > 0.05), and problem-focused (*b* = −0.02, *p* > 0.05) coping strategies. Additionally, being in the same-gender dyad was not significantly associated with adolescents’ use of active (*b* = −0.03, *p* > 0.05), distraction (*b* = 0.03, *p* > 0.05), and problem-focused (*b* = 0.01, *p* > 0.05) coping strategies.

Regarding covariates in the context of mother–adolescent communication (Table 14, Table 15 and Table 16), it was found that sibling relationship quality at Time 1 was significantly and negatively associated with the adolescents’ use of distraction (*b* = −0.06, *p* < 0.05) and problem-focused (*b* = −0.06, *p* < 0.05) coping strategies, but not with active coping (*b* = −0.05, *p* > 0.05). However, the trend was reversed at Time 2, in that sibling relationship quality was significantly and positively associated with adolescents’ use of active (*b* = 0.17, *p* < 0.05), distraction (*b* = 0.11, *p* < 0.05), and problem-focused (*b* = 0.16, *p* < 0.05) coping strategies.

Identifying as Black or African American was not significantly associated with any of the three coping strategies: active (*b* = −0.03, *p* > 0.05), distraction (*b* = 0.03, *p* > 0.05), and problem-focused (*b* = −0.02, *p* > 0.05). However, identifying as Hispanic or Latino was significantly and negative associated with adolescent’s use of active (*b* = −0.30, *p* < 0.05) and problem-focused (*b* = −0.28, *p* < 0.05), coping strategies, but not with distraction coping (*b* = −0.14, *p* > 0.05). Additionally, identifying as belonging to other racial category was not significantly associated with any of the three coping strategies: active (*b* = −0.03, *p* > 0.05), distraction (*b* = −0.07, *p* > 0.05), and problem-focused (*b* = −0.03, *p* > 0.05). Similarly, age was not associated with adolescents’ use of active (*b* = 0.05, *p* > 0.05), distraction (*b* = −0.00, *p* > 0.05), and problem-focused (*b* = 0.04, *p* > 0.05) coping strategies (Table 14, Table 15 and Table 16).

Results of the moderation analyses (Table 14, Table 15 and Table 16) revealed that in the context of mother–adolescent communication, the interaction between being in the same-gender dyad and when the participant was younger was not significant for any of the three coping strategies: active (*b* = −0.14, *p* > 0.05), distraction (*b* = −0.08, *p* > 0.05) and problem-focused (*b* = −0.17, *p* > 0.05).

For the father–adolescent communication, testing for direct effects (Table 17, Table 18 and Table 19) revealed that being younger was not significantly associated with the adolescents’ use of active (*b* = −0.09, *p* > 0.05), distraction (*b* = −0.10, *p* > 0.05), and problem-focused (*b* = −0.10, *p* > 0.05) coping strategies. Regarding gender dyads, being in the same-gender dyad was not significantly associated with the adolescents’ use of active (*b* = −0.04, *p* > 0.05), distraction (*b* = 0.03, *p* > 0.05), and problem-focused (*b* = −0.00, *p* > 0.05) coping strategies.

Furthermore, sibling relationship quality at Time 1 was not significantly associated with adolescents’ use of any of the three coping strategies (Table 17, Table 18 and Table 19): active (*b* = −0.02, *p* > 0.05), distraction (*b* = −0.04, *p* > 0.05), and problem-focused (*b* = −0.03, *p* > 0.05). However, sibling relationship quality at Time 2 was significantly and positively associated with adolescents’ use of active (*b* = 0.14, *p* < 0.05), distraction (*b* = 0.09, *p* < 0.05), and problem-focused (*b* = 0.13, *p* < 0.05) coping strategies.

Additionally, identifying as Black or African American was not significantly associated with any of the three coping strategies: active (*b* = −0.00, *p* > 0.05), distraction (*b* = 0.05, *p* > 0.05), and problem-focused (*b* = 0.00, *p* > 0.05). However, identifying as Hispanic or Latino was significantly and negatively associated with the adolescent’s use of active (*b* = −0.19, *p* < 0.05) and problem-focused coping (*b* = −0.18, *p* < 0.05), but not with distraction (*b* = −0.07, *p* < 0.05) coping. Furthermore, identifying as belonging to other racial categories was not significantly associated with adolescent’s use of any of the three coping strategies: active (*b* = 0.02, *p* > 0.05), distraction (*b* = −0.04, *p* > 0.05), and problem-focused (*b* = 0.03, *p* > 0.05). Similarly, age was not significantly associated with adolescents’ use of active (*b* = 0.02, *p* > 0.05), distraction (*b* = −0.02, *p* > 0.05), and problem-focused (*b* = 0.01, *p* > 0.05) coping strategies (Table 17, Table 18 and Table 19).

The interaction (Table 17, Table 18 and Table 19) between being in the same-gender dyad and when the participant was younger was not significant for any of the three coping strategies: active (*b* = −0.08, *p* > 0.05), distraction (*b* = −0.04, *p* > 0.05) and problem-focused (*b* = −0.10, *p* > 0.05).

**Mixed-gender dyad.** According to Table 20, Table 21, Table 22, Table 23, Table 24 and Table 25, when the participant was younger than the sibling and they were in a mixed-gender dyad, mother–adolescent communication was significantly and positively associated with adolescents’ use of active (*b* = 0.01, *p* < 0.05), distraction (*b* = 0.01, *p* < 0.05), and problem-focused (*b* = 0.01, *p* < 0.05) coping strategies. Similarly, father–adolescent communication was significantly and positively associated with the adolescents’ use of all three coping strategies: active (*b* = 0.02, *p* < 0.05), distraction (*b* = 0.01, *p* < 0.05), and problem-focused (*b* = 0.02, *p* < 0.05).

Testing for direct effects of birth order in the context of mother–adolescent communication (Table 20, Table 21 and Table 22) revealed that being younger than their sibling was significantly and negatively associated with adolescents’ use of distraction (*b* = −0.14, *p* < 0.05) and problem-focused (*b* = −0.19, *p* < 0.05) coping strategies, but not with active coping (*b* = −0.16, *p* > 0.05). Furthermore, being in a mixed-gender dyad was not significantly associated with the adolescents’ use of any of the three coping strategies: active (*b* = 0.03, *p* > 0.05), distraction (*b* = −0.03, *p* > 0.05), and problem-focused (*b* = −0.01, *p* > 0.05).

Regarding covariates (Table 20, Table 21 and Table 22), in the context of mother–adolescent communication, it was found that sibling relationship quality at Time 1 was significantly and negatively associated with adolescents’ use of distraction (*b* = −0.06, *p* < 0.05), and problem-focused (*b* = −0.06, *p* < 0.05) coping strategies, but not with active coping (*b* = −0.05, *p* > 0.05). Conversely, sibling relationship quality at Time 2 was significantly and positively associated with adolescents’ use of all three coping strategies: active (*b* = 0.17, *p* < 0.05), distraction (*b* = 0.11, *p* < 0.05), and problem-focused (*b* = 0.16, *p* < 0.05).

Furthermore, identifying as Black or African American was not significantly associated with any of the three coping strategies: active (*b* = −0.03, *p* > 0.05), distraction (*b* = −0.03, *p* > 0.05), and problem-focused (*b* = −0.03, *p* > 0.05). However, identifying as Hispanic or Latino was significantly and negatively associated with the adolescent’s use of active (*b* = −0.30, *p* < 0.05) and problem-focused (*b* = −0.28, *p* < 0.05) coping, but not with distraction coping (*b* = −0.14, *p* > 0.05). Additionally, identifying as belonging to other racial categories was not significantly associated with any of the three coping strategies: active (*b* = −0.03, *p* > 0.05), distraction (*b* = −0.07, *p* > 0.05), and problem-focused (*b* = −0.03, *p* > 0.05). Similarly, age was not significantly associated with active (*b* = 0.05, *p* > 0.05), distraction (*b* = −0.00, *p* > 0.05), and problem-focused (*b* = 0.04, *p* > 0.05) coping strategies (Table 20, Table 21 and Table 22).

In the context of mother–adolescent communication, the interaction between birth order and gender dyad was not significant for any of the three coping strategies (Table 20, Table 21 and Table 22): active (*b* = 0.14, *p* > 0.05), distraction (*b* = 0.08, *p* > 0.05), and problem-focused (*b* = 0.17, *p* > 0.05).

Upon testing for direct effects in the context of father–adolescent communication (Table 23, Table 24 and Table 25), it was found that being younger than the sibling was significantly and negatively associated with the adolescents’ use of active (*b* = −0.18, *p* < 0.05), distraction (*b* = −0.15, *p* < 0.05), and problem-focused (*b* = −0.20, *p* < 0.05) coping strategies. Additionally, being in a mixed-gender dyad was not significantly associated with the adolescents’ use of any of the three coping strategies: active (*b* = 0.04, *p* > 0.05), distraction (*b* = −0.03, *p* > 0.05), and problem-focused (*b* = 0.00, *p* > 0.05).

Additionally, regarding covariates in the context of father–adolescent communication (Table 23, Table 24 and Table 25), the findings were such that sibling relationship quality at Time 1 was not significantly associated with any of the three coping strategies: active (*b* = −0.02, *p* > 0.05), distraction (*b* = −0.04, *p* > 0.05), and problem-focused (*b* = −0.03, *p* > 0.05). However, sibling relationship at Time 2 was significantly and positively associated with adolescents’ use of active (*b* = 0.14, *p* < 0.05), distraction (*b* = 0.09, *p* < 0.05), and problem-focused (*b* = 0.13, *p* < 0.05) coping strategies.

Lastly, identifying as Black or African American was not significantly associated with any of the three coping strategies: active (*b* = −0.00, *p* > 0.05), distraction (*b* = 0.05, *p* > 0.05), and problem-focused (*b* = 0.00, *p* > 0.05). However, identifying as Hispanic or Latino was significantly and negatively associated with adolescent’s use of active (*b* = −0.19, *p* < 0.05) and problem-focused (*b* = −0.18, *p* < 0.05) coping strategy, but not with distraction coping (*b* = −0.07, *p* < 0.05). Additionally, identifying as belonging to other racial categories was not associated with any of the three coping strategies: active (*b* = 0.02, *p* > 0.05), distraction (*b* = −0.04, *p* > 0.05), and problem-focused (*b* = 0.03, *p* > 0.05). Participants’ age at Time 1 was also not significantly associated with their use of active (*b* = 0.02, *p* > 0.05), distraction (*b* = −0.02, *p* > 0.05), and problem-focused (*b* = 0.02, *p* > 0.05) coping strategies (Table 23, Table 24 and Table 25).

Similarly, in the context of father–adolescent communication, testing for moderation effects revealed no significant interaction between birth order and gender dyad for any of the three coping strategies (Table 23, Table 24 and Table 25): active (*b* = 0.08, *p* > 0.05), distraction (*b* = 0.04, *p* > 0.05), and problem-focused (*b* = 0.10, *p* > 0.05).

## 4. Discussion

Previous literature suggests siblings play a crucial role in adolescent development. According to researchers, this role extends beyond the quality of sibling relationships to their birth order and gender dyad (e.g., Barroso, 2011; Feinberg et al., 2012). Therefore, the present study aimed to examine whether birth order and gender dyad were significantly associated with the adolescents’ coping strategies- active, distraction and problem-focused. Furthermore, it also examined whether the association between birth order and adolescents’ use of three coping strategies (active, distraction, and problem-focused) is moderated by gender dyad, including mother and father–adolescent communication, sibling relationship quality from Time 1 and Time 2, adolescents’ age from Time 1, and their race and ethnicity from Time 1 as covariates.

### 4.1. Preliminary Findings

According to findings, adolescents used more active and problem-focused coping strategies than distraction. These results were partially supported by the findings of Skinner and Zimmer-Gembeck (2007), who found higher reports of problem-focused and distraction coping strategies during adolescence.

Additionally, adolescents reported lower quality of sibling relationships at Time 2 than at Time 1. This finding was unsurprising since literature suggests that the conflict between siblings peaked during early adolescence and then subsided as they transitioned to middle adolescence (Kim et al., 2006).

Furthermore, adolescents also reported better communication with mothers as compared to fathers. This finding resonates with the findings of Shek (2000), which suggested that mothers are more likely to communicate emotionally and frequently with adolescents. Consistent with this, Pantaleao and Ohannessian (2019) found that the reports of mother–adolescent communication were higher among adolescents than father–adolescent communication.

### 4.2. Hypotheses Testing

According to the results, parental communication (separately, for mothers and fathers) was positively associated with the adolescents’ use of active, problem-focused, and distraction coping strategies. This finding was consistent across all eight models, indicating that a better quality of parent–adolescent communication was associated with higher use of the three coping strategies. Pantaleao and Ohannessian (2019) found similar results, such that better mother–adolescent and father–adolescent communication was associated with a better use of adolescent coping strategies.

Findings related to the birth order were mixed, such that when the adolescent and their sibling were in the same-gender dyad, findings were such that participants’ birth order was not significantly associated with their use of any of the three coping strategies for either mothers’ or fathers’ communication. Specifically, being older or younger than their sibling was not significantly associated with the adolescents’ use of any of the three strategies: active, problem-focused, and distraction. On the contrary, when the participant and their sibling were in a mixed-gender dyad, birth order was significantly associated with the adolescents’ use of active, distraction, and problem-focused coping strategies, for both mothers’ and fathers’ communication. Specifically, being older than the sibling increased the likelihood of the adolescent’s use of the three coping strategies. This finding is novel to the present study, in that, to the authors’ knowledge no previous literature has established this association. Furthermore, as posited in the family systems theory, older siblings often play the role of “*hero*” for their younger siblings (Smith & Hamon, 2022). In this case, older siblings might model using coping strategies for their younger siblings who are still developing these resources.

Similarly, findings regarding the gender dyads revealed mixed results. When the participant was older, gender dyad was significantly associated with the adolescents’ use of active and problem-focused coping strategies but not distraction for both mothers and fathers communication. The findings were such that being of the same-gender dyad decreased the likelihood of adolescents using active and problem-focused coping strategies but not distraction coping. However, when the participant was younger, gender dyad was not significantly associated with the adolescents’ use of any of the three coping strategies in either mothers’ or fathers’ communication.

Discrepancies in findings may be attributed to the unique experiences of adolescents based on their birth order and gender dyad. The quality of sibling relationships has been found to vary based on the gender dyad. For example, Campione-Barr and Smetana (2010) found that siblings in the same-gender dyad had higher levels of communication as compared to those in a mixed-gender dyad. Similarly, Law and Imran (2024) found that same-gender siblings, specifically when both the adolescent and their sibling were female, reported a better perception of the sibling relationship. Sibling relationship quality, in turn, has been found to predict adolescent psychological adjustment. For example, in their meta-analysis, Buist et al. (2013) found that high sibling warmth and less sibling conflict were associated with lower internalizing and externalizing problems. Furthermore, studies regarding birth order and adolescent psychological adjustment have yielded mixed results as well. For example, İşgör and Özpolat (2017) found that middle children reported higher safe attachment as compared to those who were single children. Conversely, Clayton et al. (2023) found no significant association between adolescents’ birth order and their attachment style.

According to the family systems theory, *family roles* are influenced by the hierarchy of family members (Smith & Hamon, 2022). Particularly among siblings, the older child is often referred to as the *hero* who is the ideal caretaker and student (Smith & Hamon, 2022). This perceived responsibility might create pressure on the adolescent to demonstrate functional behavior and coping mechanisms for their younger siblings, who might admire them and behave in accordance with their example.

Interestingly, the association between sibling relationship quality and adolescents’ use of the three coping strategies varied on the time point at which the data were collected Particularly, sibling relationship quality at Time 1 was significantly and negatively associated with adolescents’ use of all three coping strategies: active, distraction and problem-focused. However, the trend was reversed at Time 2 where sibling relationship quality at Time 2 was significantly and positively associated with the three coping strategies. This longitudinal reversal may reflect the change in the functional role of sibling relationship. At Time 1, a high-quality sibling relationship might serve as an environmental shield, mitigating stress and reducing the need for coping strategies. However, at Time 2, the same relationship quality might act as a socialization mechanism, promoting the development of adolescent coping strategies.

The results of the interaction revealed that participants being older significantly interacted with gender dyad, and for both mothers’ and fathers’ communication. 

**Figure 1:**
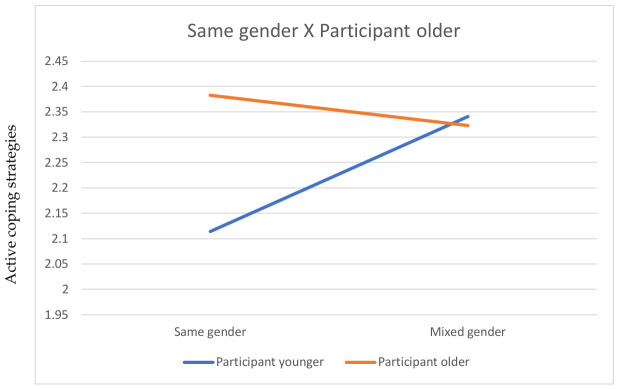
Model for mothers’ communication. *Note:* outcome = active coping strategy.

For mother–adolescent communication, the significant interaction between birth order (participant older) and sibling gender dyad (same-gender) on active coping strategies was probed by calculating discrete, cell-specific predicted means, holding all background covariates constant at their sample averages. As shown in Figure 1, the pattern of active coping differed based on the interaction of birth order and dyad composition. For younger participants, those in same-gender sibling dyads reported notably lower use of active coping strategies compared to those in mixed-gender dyads. Conversely, among older participants, those in same-gender dyads reported slightly higher use of active coping strategy relative to those in mixed-gender dyads. Finally, when examining participants specifically within same-gender dyads, older siblings reported significantly higher use of active coping strategies than their younger counterparts.

**Figure 2:**
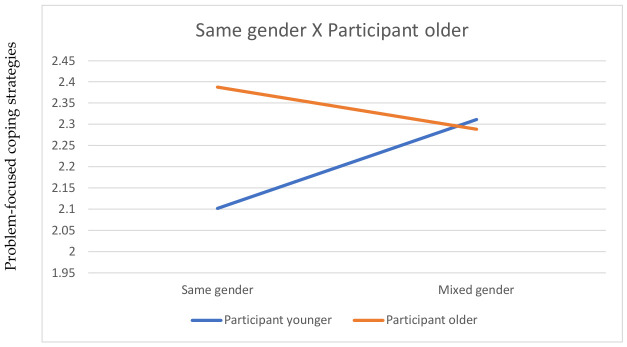
*Model* for mothers’ communication. *Note:* outcome = problem-focused coping strategy.

Similarly, for problem-focused coping, the significant interaction was probed by calculating discrete, cell-specific predicted means, holding all background covariates constant at their sample averages. As shown in Figure 2, the pattern of problem-focused coping differed based on the interaction of birth order and dyad composition. For younger participants, those in same-gender sibling dyads reported lower use of problem-focused coping strategies compared to those in mixed-gender dyads. Conversely, among older participants, those in same-gender dyads reported slightly higher use of problem-focused coping strategy relative to those in mixed-gender dyads. Finally, when examining participants specifically within same-gender dyads, older siblings reported significantly higher use of problem-focused coping strategies than their younger counterparts.

**Figure 3:**
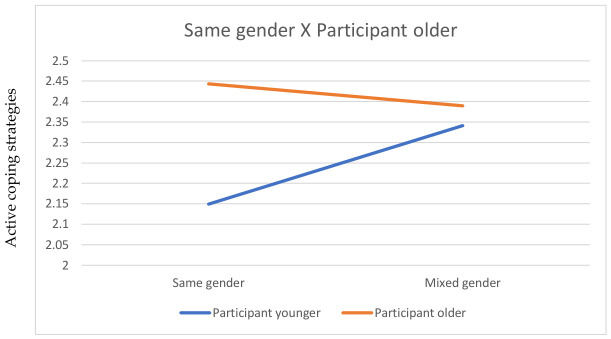
Model for fathers’ communication. *Note:* outcome = active coping strategy.

For active coping strategies, in father–adolescent communication, significant interaction term between the same-gender dyad and when the participant was older was probed by calculating discrete, cell-specific predicted means, holding all background covariates constant at their sample averages. As shown in Figure 3, the pattern of active coping differed based on the interaction of birth order and dyad composition. For younger participants, those in same-gender sibling dyads reported notably lower use of active coping strategies compared to those in mixed-gender dyads. Conversely, among older participants, those in same-gender dyads reported slightly higher use of active coping strategy relative to those in mixed-gender dyads. Finally, when examining participants specifically within same-gender dyads, older siblings reported significantly higher use of active coping strategies than their younger counterparts.

**Figure 4:**
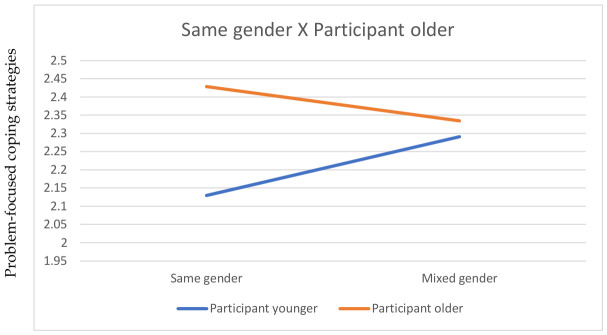
Model for fathers’ communication. *Note:* outcome = problem-focused coping strategy.

Additionally, the significant interaction term for the problem-focused coping strategy was probed by calculating discrete, cell-specific predicted means, holding all background covariates constant at their sample averages. As shown in Figure 4, the pattern of problem-focused coping differed based on the interaction of birth order and dyad composition. For younger participants, those in same-gender sibling dyads reported notably lower use of problem-focused coping strategies compared to those in mixed-gender dyads. Conversely, among older participants, those in same-gender dyads reported slightly higher use of problem-focused coping strategy relative to those in mixed-gender dyads. Finally, when examining participants specifically within same-gender dyads, older siblings reported significantly higher use of problem-focused coping strategies than their younger counterparts.

**Figure 5:**
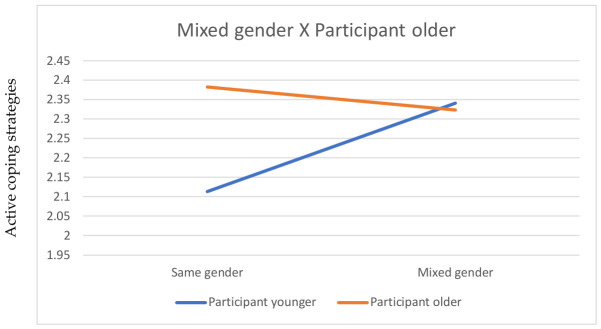
Model for mothers’ communication. *Note:* outcome = active coping strategy.

Significant interactions when the participant was older and in a mixed-gender dyad for mother–adolescent communication was probed by calculating discrete, cell-specific predicted means, holding all background covariates constant at their sample averages. As shown in Figure 5, the pattern of active coping differed based on the interaction of birth order and dyad composition. For younger participants, those in same-gender sibling dyads reported notably lower use of active coping strategies compared to those in mixed-gender dyads. Conversely, among older participants, those in same-gender dyads reported slightly higher use of active coping strategy relative to those in mixed-gender dyads. Finally, when examining participants specifically within same-gender dyads, older siblings reported significantly higher use of active coping strategies than their younger counterparts.

**Figure 6:**
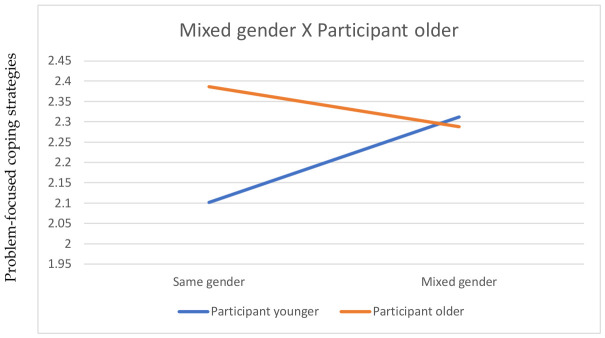
Model for mothers’ communication. *Note:* outcome = problem-focused coping strategy.

Additionally, the significant interaction term for problem-focused coping strategies was probed by calculating discrete, cell-specific predicted means, holding all background covariates constant at their sample averages. As shown in Figure 6, the pattern of problem-focused coping differed based on the interaction of birth order and dyad composition. For younger participants, those in same-gender sibling dyads reported notably lower use of problem-focused coping strategies compared to those in mixed-gender dyads. Conversely, among older participants, those in same-gender dyads reported slightly higher use of problem-focused coping strategy relative to those in mixed-gender dyads. Finally, when examining participants specifically within same-gender dyads, older siblings reported significantly higher use of problem-focused coping strategies than their younger counterparts.

**Figure 7:**
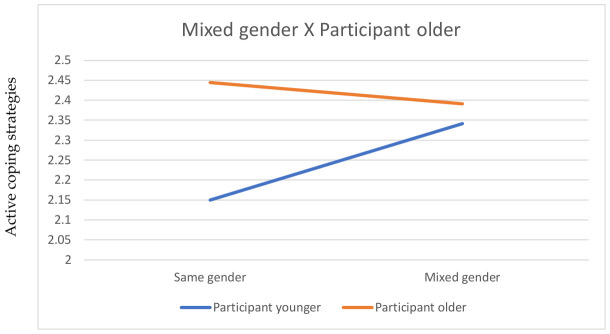
Model for fathers’ communication. *Note:* outcome = active coping strategy.

Furthermore, for father–adolescent communication, the significant interaction term between when the participant was older and in a mixed-gender dyad for active coping strategies was probed by calculating discrete, cell-specific predicted means, holding all background covariates constant at their sample averages. As shown in Figure 7, the pattern of active coping differed based on the interaction of birth order and dyad composition. For younger participants, those in same-gender sibling dyads reported notably lower use of active coping strategies compared to those in mixed-gender dyads. Conversely, among older participants, those in same-gender dyads reported slightly higher use of active coping strategy relative to those in mixed-gender dyads. Finally, when examining participants specifically within same-gender dyads, older siblings reported significantly higher use of active coping strategies than their younger counterparts.

**Figure 8:**
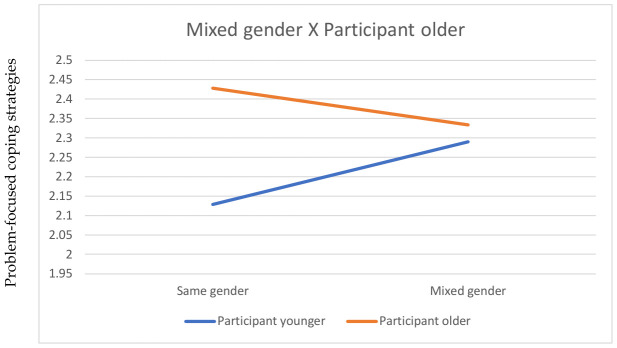
Model for fathers’ communication. *Note:* outcome = problem-focused coping strategy.

Significant interaction for the outcome problem-focused coping was probed by calculating discrete, cell-specific predicted means, holding all background covariates constant at their sample averages. As shown in Figure 8, the pattern of problem-focused coping differed based on the interaction of birth order and dyad composition. For younger participants, those in same-gender sibling dyads reported notably lower use of problem-focused coping strategies compared to those in mixed-gender dyads. Conversely, among older participants, those in same-gender dyads reported slightly higher use of problem-focused coping strategy relative to those in mixed-gender dyads. Finally, when examining participants specifically within same-gender dyads, older siblings reported significantly higher use of problem-focused coping strategies than their younger counterparts.

To summarize, model testing for birth order and gender dyad in the context of mothers’ and fathers’ communication revealed a pattern. Particularly, older participants reported higher use of active and problem-focused coping across all eight interactions and in the context of mothers’ and father’s communication. Furthermore, younger participants in a mixed-gender dyad reported higher use of both active and problem-focused coping as compared to the ones in the same-gender dyad, whereas older participants in the mixed-gender dyad reported lower use of the two coping strategies as compared to the ones in same-gender dyad.

The results of interaction effects from mothers and fathers’ communication can be understood in the context of the family systems theory, particularly the presence of subsystems within a family system (Smith & Hamon, 2022). According to the theory, the various subsystems, even though they are interrelated, have unique characteristics of their own. These characteristics can be based on the individual features of the members of the subsystem or on their circumstances. For example, even though both mother–adolescent and father–adolescent subsystems are nested within the larger family system, they still retain their unique properties. Particularly, mother–adolescent communication is characterized by higher emotion and frequency, as compared to father–adolescent communication (Shek, 2000). These differences in patterns of communication can have inherently different outcomes for adolescent development. Therefore, the varying results of the interaction effects in the context of mother and father communication further highlight the uniqueness of the subsystems, including each parental relationship separately rather than as a single construct.

The significant interactions highlight that, along with the quality of sibling relationship, the dynamics of this dyad are also significant in determining the developmental outcomes among adolescents. Previous studies have found similar results where they established that sibling disclosure varied by birth order and gender dyad (Zhou et al., 2024). Additionally, Adamov et al. (2024) found that the perception of family environment and its influence on adolescents’ life satisfaction varied based on their birth order and gender dyad, with the association between family support and life satisfaction being stronger for same-gender dyads and those where the brothers were older.

One notable demographic characteristic of the sample was the wide age range of siblings, ranging from infancy to older adulthood. Particularly, one of the participants reported that the sibling closest to them in age was 60 years old. While the traditional developmental models on siblings focus on dyads closer in age (for example, McHale et al., 2012) a wider age range is more reflective of a blended family structure with children from previous unions (Cherlin, 2010). In such cases, substantially older siblings are more likely seen as quasi-parent rather than as siblings and the dynamic relationship mirrors the parent–adolescent relationship.

The present study presents some limitations. First, the data used in this study were collected in the Northeast region of the United States, limiting its generalizability to other regions and populations. Second, the study relied on self-report data from adolescents. Given the nature of the variables, parent–adolescent communication and sibling relationship quality, it is crucial to assess how these variables are perceived by their parents and siblings. Additionally, the scale for adolescents’ coping strategies presented a lower alpha, possibly affecting the results. Furthermore, most of the participants in the study identified themselves as White, thus further limiting its applicability to individuals from other cultures and ethnicities. Lastly, even though the present study found significant effects, the magnitude of the effect sizes was small (ranging from 0.00 to 0.04), limiting the applicability of the results.

However, despite the limitations, the present study also has strong implications for family and child researchers and practitioners. Using this study as background literature, future studies can further probe into the reasons for its varying results. Future studies can also examine whether there are other factors, such as the presence of multiple siblings and the age gap between siblings play a role in adolescents’ development of coping strategies. Furthermore, a diverse sample, in terms of diverse culture and socioeconomic status can increase the generalizability of the findings. And finally, using reports from multiple sources such as from parents and siblings, future studies can examine whether they obtain similar results.

Regarding practitioners, given the small effect sizes, utilizing these results, particularly regarding the role of parent–adolescent communication in therapy, should treat it as a clinical supplement, rather than as a primary determinant of adolescent coping strategies. Child and family therapists can leverage the presence of siblings in the household to improve the adolescents’ coping strategies by incorporating sibling-inclusive therapy sessions and recognizing them as active participants in the family system. Similarly, the present study also highlights the importance of training practitioners working with at-risk youth to include sibling relationship assessment as a routine screening tool and also assessing the gender and birth order of the sibling. Additionally, the school counselors and therapists can develop a psychoeducational session for adolescents to teach them adaptive coping strategies, particularly in their family environment. Furthermore, after identifying the signs of maladaptive coping strategies, practitioners, such as school counselors, can assess the underlying issues present within the family system and design interventions tailored to address those issues to promote adaptive coping among adolescents. Lastly, for practitioners designing family and educational interventions, this study can be useful to focus on not just the presence of siblings but also their birth order and gender with respect to the client to assess their influence on the development of adaptive coping strategies.

## Figures and Tables

**Table 1:** Summary and descriptive statistics of study variables.

Variables	Mean/%	SD	Minimum	Maximum
Active coping (T3)	2.35	0.77	1	4
Distraction coping (T3)	2.19	0.61	1	4
Problem-focused coping (T3)	2.35	0.77	1	4
Mother–adolescent communication (T1)	71.08	14.50	27	100
Father–adolescent communication (T1)	70.18	15.18	25	100
Sibling support (T1)	4.82	1.55	1	7
Sibling support (T2)	4.74	1.60	1	7
Participant’s age (T1)	12.75	0.71	11	15
Participant’s age (T2)	13.11	0.79	11	17
Sibling’s age (T2)	13.73	5.30	0	60
Participant’s Gender (T1)				
Male	48.51			
Female	51.49			
Sibling’s Gender (T2)				
Male	48.89			
Female	51.11			
Race (T1)				
White	52.15			
Black/African American	8.75			
Hispanic/Latino	19.88			
Other	19.22			
Birth order				
Older	42.86			
Younger	57.14			
Gender dyad				
Same-gender	51.80			
Mixed-gender	48.20			

**Table 2:** Path coefficients from predictors, interaction terms and covariates to the outcome.

Variables	*b*	*SE*	95% CI		*p*
			*LL*	*UL*	
Mother–adolescent communication (T1)	0.01	0.00	0.00	0.01	0.000 **
Participant older	−0.02	0.09	−0.20	0.16	0.843
Same-gender dyad	−0.23	0.08	−0.39	−0.06	0.006 **
OlderXSame-gender dyad	0.29	0.13	0.03	0.54	0.026 *
Sibling relationship quality (T1)	−0.05	0.02	−0.09	−0.00	0.055
Sibling relationship quality (T2)	0.17	0.02	0.13	0.21	0.000 **
Participant’s race—Black or African American (T1)	−0.04	0.13	−0.29	0.21	0.757
Participant’s race—Hispanic or Latino (T1)	−0.30	0.10	−0.50	−0.11	0.002 **
Participant’s race—other (T1)	−0.04	0.08	−0.19	0.11	0.621
Participant’s age (T1)	0.04	0.04	−0.04	0.13	0.305

*Note. N* = 1428, CI = confidence interval, LL = lower limit, UL = upper limit, * *p* < 0.05, ** *p* < 0.01, outcome = active coping strategies (T3).

**Table 3:** Path coefficients from predictors, interaction terms and covariates to the outcome.

Variables	*b*	*SE*	95% CI		*p*
			*LL*	*UL*	
Mother–adolescent communication (T1)	0.01	0.00	0.00	0.01	0.000 **
Participant older	0.05	0.07	−0.10	0.19	0.474
Same-gender dyad	−0.08	0.07	−0.21	0.04	0.212
OlderXSame-gender dyad	0.17	0.10	−0.03	0.37	0.101
Sibling relationship quality (T1)	−0.06	0.02	−0.10	−0.02	0.002 **
Sibling relationship quality (T2)	0.11	0.02	0.08	0.14	0.000 **
Participant’s race—Black or African American (T1)	−0.03	0.10	−0.17	0.23	0.774
Participant’s race—Hispanic or Latino (T1)	−0.14	0.07	−0.29	0.00	0.057
Participant’s race—other (T1)	−0.08	0.06	−0.20	0.04	0.216
Participant’s age (T1)	0.00	0.03	−0.07	0.07	0.959

*Note. N* = 1428, CI = confidence interval, LL = lower limit, UL = upper limit, ** *p* < 0.01, outcome= distraction coping strategies (T3).

**Table 4:** Path coefficients from predictors, interaction terms and covariates to the outcome.

Variables	*b*	*SE*	95% CI		*p*
			*LL*	*UL*	
Mother–adolescent communication (T1)	0.01	0.00	0.01	0.01	0.000 **
Participant older	−0.02	0.09	−0.20	0.15	0.794
Same-gender dyad	−0.21	0.08	−0.37	−0.05	0.010 *
OlderXSame-gender dyad	0.31	0.13	0.06	0.56	0.015 *
Sibling relationship quality (T1)	−0.06	0.02	−0.10	−0.01	0.019 **
Sibling relationship quality (T2)	0.16	0.02	0.12	0.10	0.000 **
Participant’s race—Black or African American (T1)	−0.04	0.12	0.28	0.20	0.769
Participant’s race—Hispanic or Latino (T1)	−0.29	0.09	−0.47	−0.10	0.002 **
Participant’s race—other (T1)	−0.04	0.08	−0.18	0.12	0.682
Participant’s age (T1)	0.04	0.04	−0.05	0.12	0.387

*Note. N* = 1428, CI = confidence interval, LL = lower limit, UL = upper limit, * *p* < 0.05, ** *p* < 0.01, outcome = problem-focused coping strategies (T3).

**Table 5:** Path coefficients from predictors, interaction terms and covariates to the outcome.

Variables	*b*	*SE*	95% CI		*p*
			*LL*	*UL*	
Father–adolescent communication (T1)	0.02	0.00	0.02	0.02	0.000 **
Participant older	0.05	0.09	−0.12	0.22	0.584
Same-gender dyad	−0.19	0.07	−0.34	−0.03	0.014 *
OlderXSame-gender dyad	0.25	0.12	0.01	0.49	0.043 *
Sibling relationship quality (T1)	−0.02	0.02	−0.07	0.02	0.305
Sibling relationship quality (T2)	0.14	0.02	0.09	0.18	0.000 **
Participant’s race—Black or African American (T1)	−0.00	0.12	−0.24	0.24	0.986
Participant’s race—Hispanic or Latino (T1)	−0.20	0.09	−0.38	−0.02	0.031 *
Participant’s race—other (T1)	0.01	0.07	−0.13	0.15	0.882
Participant’s age (T1)	0.02	0.04	−0.06	0.10	0.622

*Note. N* = 1428, CI = confidence interval, LL = lower limit, UL = upper limit, * *p* < 0.05, ** *p* < 0.01, outcome = active coping strategies (T3).

**Table 6:** Path coefficients from predictors, interaction terms and covariates to the outcome.

Variables	*b*	*SE*	95% CI		*p*
			*LL*	*UL*	
Father–adolescent communication (T1)	0.01	0.00	0.01	0.02	0.000 **
Participant older	0.09	0.07	−0.05	0.23	0.210
Same-gender dyad	−0.05	0.06	−0.18	0.07	0.423
OlderXSame-gender dyad	0.13	0.10	−0.06	0.33	0.178
Sibling relationship quality (T1)	−0.04	0.02	−0.07	0.00	0.049 *
Sibling relationship quality (T2)	0.08	0.01	0.05	0.12	0.000 **
Participant’s race—Black or African American (T1)	−0.05	0.10	−0.14	0.25	0.609
Participant’s race—Hispanic or Latino (T1)	−0.07	0.07	−0.22	0.07	0.314
Participant’s race—other (T1)	−0.05	0.06	−0.17	0.07	0.393
Participant’s age (T1)	−0.01	0.03	−0.08	0.05	0.612

*Note. N* = 1428, CI = confidence interval, LL = lower limit, UL = upper limit, * *p* < 0.05, ** *p* < 0.01, outcome = distraction coping strategies (T3).

**Table 7:** Path coefficients from predictors, interaction terms and covariates to the outcome.

Variables	*b*	*SE*	95% CI		*p*
			*LL*	*UL*	
Father–adolescent communication (T1)	0.02	0.00	0.02	0.02	0.000 **
Participant older	0.04	0.09	−0.13	0.22	0.612
Same-gender dyad	−0.16	0.08	−0.31	−0.01	0.038 *
OlderXSame-gender dyad	0.25	0.12	0.02	0.49	0.035 *
Sibling relationship quality (T1)	−0.04	0.02	−0.08	0.01	0.114
Sibling relationship quality (T2)	0.13	0.02	0.08	0.17	0.000 **
Participant’s race—Black or African American (T1)	−0.00	0.12	−0.23	0.23	0.979
Participant’s race—Hispanic or Latino (T1)	−0.19	0.10	−0.37	−0.01	0.037 *
Participant’s race—other (T1)	0.02	0.07	−0.12	0.16	0.766
Participant’s age (T1)	0.02	0.04	−0.06	0.10	0.646

*Note. N* = 1428, CI = confidence interval, LL = lower limit, UL = upper limit, * *p* < 0.05, ** *p* < 0.01, outcome = problem-focused coping strategies (T3).

**Table 8:** Path coefficients from predictors, interaction terms and covariates to the outcome.

Variables	*b*	*SE*	95% CI		*p*
			*LL*	*UL*	
Mother–adolescent communication (T1)	0.01	0.00	0.01	0.01	0.000 **
Participant older	0.27	0.08	0.10	0.44	0.002 **
Mixed-gender dyad	0.23	0.08	0.06	0.39	0.006 **
OlderXMixed-gender dyad	−0.29	0.13	−0.54	−0.03	0.026 *
Sibling relationship quality (T1)	−0.05	0.02	−0.10	−0.00	0.055
Sibling relationship quality (T2)	0.17	0.02	0.13	0.21	0.000 **
Participant’s race—Black or African American (T1)	−0.04	0.13	−0.29	0.21	0.757
Participant’s race—Hispanic or Latino (T1)	−0.30	0.10	−0.49	−0.11	0.002 **
Participant’s race—other (T1)	−0.04	0.08	−0.19	0.11	0.621
Participant’s age (T1)	0.04	0.04	−0.04	0.13	0.305

*Note. N* = 1428, CI = confidence interval, LL = lower limit, UL = upper limit, * *p* < 0.05, ** *p* < 0.01, outcome = active coping strategies (T3).

**Table 9:** Path coefficients from predictors, interaction terms and covariates to the outcome.

Variables	*b*	*SE*	95% CI		*p*
			*LL*	*UL*	
Mother–adolescent communication (T1)	0.01	0.00	0.00	0.01	0.000 **
Participant older	0.22	0.06	0.09	0.35	0.001 **
Mixed-gender dyad	0.08	0.06	−0.05	0.21	0.212
OlderXMixed-gender dyad	−0.17	0.10	−0.37	0.03	0.101
Sibling relationship quality (T1)	−0.06	0.02	−0.10	−0.02	0.002 **
Sibling relationship quality (T2)	0.11	0.02	0.08	0.14	0.000 **
Participant’s race—Black or African American (T1)	−0.03	0.10	−0.17	0.23	0.774
Participant’s race—Hispanic or Latino (T1)	−0.14	0.07	−0.29	0.00	0.057
Participant’s race—other (T1)	−0.08	0.06	−0.20	0.04	0.216
Participant’s age (T1)	−0.00	0.03	−0.07	0.07	0.959

*Note. N* = 1428, CI = confidence interval, LL = lower limit, UL = upper limit, ** *p* < 0.01, outcome = distraction coping strategies (T3).

**Table 10:** Path coefficients from predictors, interaction terms and covariates to the outcome.

Variables	*b*	*SE*	95% CI		*p*
			*LL*	*UL*	
Mother–adolescent communication (T1)	0.01	0.00	0.01	0.02	0.000 **
Participant older	0.28	0.08	0.12	0.45	0.001 **
Mixed-gender dyad	0.21	0.08	0.05	0.37	0.010 *
OlderXMixed-gender dyad	−0.31	0.13	−0.56	−0.06	0.015 *
Sibling relationship quality (T1)	−0.06	0.02	−0.10	−0.01	0.019 *
Sibling relationship quality (T2)	0.16	0.02	0.12	0.20	0.000 **
Participant’s race—Black or African American (T1)	−0.04	0.12	−0.28	0.21	0.769
Participant’s race—Hispanic or Latino (T1)	−0.29	0.09	−0.47	−0.10	0.002 **
Participant’s race—other (T1)	−0.03	0.08	−0.18	0.12	0.682
Participant’s age (T1)	0.04	0.04	−0.05	0.12	0.387

*Note. N* = 1428, CI = confidence interval, LL = lower limit, UL = upper limit, * *p* < 0.05, ** *p* < 0.01, outcome = problem-focused coping strategies (T3).

**Table 11:** Path coefficients from predictors, interaction terms and covariates to the outcome.

Variables	*b*	*SE*	95% CI		*p*
			*LL*	*UL*	
Father–adolescent communication (T1)	0.02	0.00	0.02	0.02	0.000 **
Participant older	0.29	0.08	0.14	0.45	0.000 **
Mixed-gender dyad	0.19	0.08	0.04	0.35	0.014 **
OlderXMixed-gender dyad	−0.25	0.12	−0.49	−0.01	0.043 *
Sibling relationship quality (T1)	−0.02	0.02	−0.07	0.02	0.305
Sibling relationship quality (T2)	0.14	0.02	0.09	0.18	0.000 **
Participant’s race—Black or African American (T1)	−0.00	0.12	−0.24	0.24	0.986
Participant’s race—Hispanic or Latino (T1)	−0.20	0.09	−0.38	−0.02	0.031 *
Participant’s race—other (T1)	0.01	0.07	−0.13	0.15	0.882
Participant’s age (T1)	0.02	0.04	−0.06	0.10	0.622

*Note. N* = 1428, CI = confidence interval, LL = lower limit, UL = upper limit, * *p* < 0.05, ** *p* < 0.01, outcome = active coping strategies (T3).

**Table 12:** Path coefficients from predictors, interaction terms and covariates to the outcome.

Variables	*b*	*SE*	95% CI		*p*
			*LL*	*UL*	
Father–adolescent communication (T1)	0.01	0.00	0.01	0.01	0.000 **
Participant older	0.23	0.06	−0.10	0.01	0.001 **
Mixed-gender dyad	0.05	0.06	−0.07	0.17	0.423
OlderXMixed-gender dyad	−0.13	0.10	−0.33	0.06	0.178
Sibling relationship quality (T1)	−0.04	0.02	−0.07	0.00	0.049 *
Sibling relationship quality (T2)	0.09	0.02	0.05	0.12	0.000 **
Participant’s race—Black or African American (T1)	0.05	0.10	−0.14	0.25	0.609
Participant’s race—Hispanic or Latino (T1)	−0.07	0.07	−0.22	0.07	0.314
Participant’s race—other (T1)	−0.05	0.06	−0.17	0.07	0.393
Participant’s age (T1)	−0.02	0.03	−0.08	0.05	0.612

*Note. N* = 1428, CI = confidence interval, LL = lower limit, UL = upper limit, * *p* < 0.05, ** *p* < 0.01, outcome = distraction coping strategies (T3).

**Table 13:** Path coefficients from predictors, interaction terms and covariates to the outcome.

Variables	*b*	*SE*	95% CI		*p*
			*LL*	*UL*	
Father–adolescent communication (T1)	0.02	0.00	0.02	0.02	0.000 **
Participant older	0.30	0.08	0.14	0.46	0.000 **
Mixed-gender dyad	0.16	0.08	0.00	0.31	0.038 *
OlderXMixed-gender dyad	−0.25	0.12	−0.49	−0.01	0.035 *
Sibling relationship quality (T1)	−0.04	0.02	−0.08	0.01	0.114
Sibling relationship quality (T2)	0.13	0.02	0.08	0.17	0.000 **
Participant’s race—Black or African American (T1)	0.00	0.12	−0.23	0.23	0.979
Participant’s race—Hispanic or Latino (T1)	−0.19	0.09	−0.37	−0.01	0.037 *
Participant’s race—other (T1)	0.02	0.07	−0.12	0.16	0.766
Participant’s age (T1)	0.02	0.04	−0.06	0.10	0.646

*Note. N* = 1428, CI = confidence interval, LL = lower limit, UL = upper limit, * *p* < 0.05, ** *p* < 0.01, outcome = problem-focused coping strategies (T3).

**Table 14:** Path coefficients from predictors, interaction terms and covariates to the outcome.

Variables	*b*	*SE*	95% CI		*p*
			*LL*	*UL*	
Mother–adolescent communication (T1)	0.01	0.00	0.01	0.02	0.000 **
Participant younger	−0.02	0.09	−0.20	0.16	0.829
Same-gender dyad	−0.03	0.09	−0.22	0.15	0.727
YoungerXSame-gender dyad	−0.14	0.13	−0.39	0.12	0.291
Sibling relationship quality (T1)	−0.05	0.02	−0.10	0.00	0.052
Sibling relationship quality (T2)	0.17	0.02	0.13	0.21	0.000 **
Participant’s race—Black or African American (T1)	−0.03	0.13	−0.28	0.22	0.808
Participant’s race—Hispanic or Latino (T1)	−0.30	0.10	−0.49	−0.11	0.002 **
Participant’s race—other (T1)	−0.03	0.08	−0.19	0.12	0.667
Participant’s age (T1)	0.05	0.04	−0.04	0.13	0.301

*Note. N* = 1428, CI = confidence interval, LL = lower limit, UL = upper limit, ** *p* < 0.01, outcome = active coping strategies (T3).

**Table 15:** Path coefficients from predictors, interaction terms and covariates to the outcome.

Variables	*b*	*SE*	95% CI		*p*
			*LL*	*UL*	
Mother–adolescent communication (T1)	0.01	0.00	0.00	0.01	0.000 **
Participant younger	−0.06	0.07	−0.21	0.08	0.394
Same-gender dyad	0.03	0.07	−0.11	0.18	0.654
YoungerXSame-gender dyad	−0.08	0.10	−0.28	0.12	0.423
Sibling relationship quality (T1)	−0.06	0.02	−0.10	−0.02	0.002 **
Sibling relationship quality (T2)	0.11	0.02	0.08	0.15	0.000 **
Participant’s race—Black or African American (T1)	0.03	0.10	−0.17	0.23	0.744
Participant’s race—Hispanic or Latino (T1)	−0.14	0.07	−0.28	0.01	0.068
Participant’s race—other (T1)	−0.07	0.06	−0.19	0.05	0.261
Participant’s age (T1)	−0.00	0.03	−0.07	0.06	0.900

*Note. N* = 1428, CI = confidence interval, LL = lower limit, UL = upper limit, ** *p* < 0.01, outcome = distraction coping strategies (T3).

**Table 16:** Path coefficients from predictors, interaction terms and covariates to the outcome.

Variables	*b*	*SE*	95% CI		*p*
			*LL*	*UL*	
Mother–adolescent communication (T1)	0.01	0.00	0.01	0.02	0.000 **
Participant younger	−0.02	0.09	−0.20	0.15	0.777
Same-gender dyad	0.01	0.09	−0.17	0.19	0.915
YoungerXSame-gender dyad	−0.17	0.13	−0.42	0.08	0.188
Sibling relationship quality (T1)	−0.06	0.02	−0.10	−0.01	0.020 *
Sibling relationship quality (T2)	0.16	0.02	0.12	0.20	0.000 **
Participant’s race—Black or African American (T1)	−0.03	0.12	−0.27	0.22	0.821
Participant’s race—Hispanic or Latino (T1)	−0.28	0.09	−0.47	0.10	0.003 **
Participant’s race—other (T1)	−0.03	0.08	−0.18	0.13	0.736
Participant’s age (T1)	0.04	0.04	−0.05	0.12	0.395

*Note. N* = 1428, CI = confidence interval, LL = lower limit, UL = upper limit, * *p* < 0.05, ** *p* < 0.01, outcome = problem-focused coping strategies (T3).

**Table 17:** Path coefficients from predictors, interaction terms and covariates to the outcome.

Variables	*b*	*SE*	95% CI		*p*
			*LL*	*UL*	
Father–adolescent communication (T1)	0.02	0.00	0.02	0.02	0.000 **
Participant younger	−0.10	0.09	−0.27	0.08	0.295
Same-gender dyad	−0.04	0.09	−0.22	0.13	0.625
YoungerXSame-gender dyad	−0.08	0.12	−0.32	0.15	0.485
Sibling relationship quality (T1)	−0.02	0.02	−0.07	0.02	0.320
Sibling relationship quality (T2)	0.14	0.02	0.10	0.18	0.000 **
Participant’s race—Black or African American (T1)	−0.00	0.12	−0.24	0.23	0.982
Participant’s race—Hispanic or Latino (T1)	−0.19	0.09	−0.37	−0.01	0.039 *
Participant’s race—other (T1)	0.02	0.07	−0.13	0.16	0.804
Participant’s age (T1)	0.02	0.04	−0.06	0.10	0.668

*Note. N* = 1428, CI = confidence interval, LL = lower limit, UL = upper limit, * *p* < 0.05, ** *p* < 0.01, outcome = active coping strategies (T3).

**Table 18:** Path coefficients from predictors, interaction terms and covariates to the outcome.

Variables	*b*	*SE*	95% CI		*p*
			*LL*	*UL*	
Father–adolescent communication (T1)	0.01	0.00	0.01	0.01	0.000 **
Participant younger	−0.11	0.07	−0.25	0.03	0.140
Same-gender dyad	0.03	0.07	−0.11	0.17	0.702
YoungerXSame-gender dyad	−0.04	0.10	−0.23	0.15	0.689
Sibling relationship quality (T1)	−0.04	0.02	−0.07	0.00	0.053
Sibling relationship quality (T2)	0.09	0.02	0.05	0.12	0.000 **
Participant’s race—Black or African American (T1)	−0.05	0.10	−0.15	0.24	0.626
Participant’s race—Hispanic or Latino (T1)	−0.07	0.07	−0.21	0.08	0.366
Participant’s race—other (T1)	0.04	0.06	−0.16	0.08	0.475
Participant’s age (T1)	−0.02	0.03	−0.09	0.04	0.521

*Note. N* = 1428, CI = confidence interval, LL = lower limit, UL = upper limit, ** *p* < 0.01, outcome = distraction coping strategies (T3).

**Table 19:** Path coefficients from predictors, interaction terms and covariates to the outcome.

Variables	*b*	*SE*	95% CI		*p*
			*LL*	*UL*	
Father–adolescent communication (T1)	0.02	0.00	0.02	0.02	0.000 **
Participant younger	−0.10	0.09	−0.27	0.07	0.245
Same-gender dyad	−0.00	0.09	−0.17	0.17	0.991
YoungerXSame-gender dyad	−0.10	0.12	−0.34	0.14	0.408
Sibling relationship quality (T1)	−0.03	0.02	−0.08	0.01	0.128
Sibling relationship quality (T2)	0.13	0.02	0.09	0.17	0.000 **
Participant’s race—Black or African American (T1)	0.00	0.12	−0.23	0.23	0.981
Participant’s race—Hispanic or Latino (T1)	−0.18	0.09	−0.36	−0.00	0.048 *
Participant’s race—other (T1)	0.03	0.07	−0.11	0.17	0.684
Participant’s age (T1)	0.01	0.04	−0.07	0.10	0.708

*Note. N* = 1428, CI = confidence interval, LL = lower limit, UL = upper limit, * *p* < 0.05, ** *p* < 0.01, outcome = problem-focused coping strategies (T3).

**Table 20:** Path coefficients from predictors, interaction terms and covariates to the outcome.

Variables	*b*	*SE*	95% CI		*p*
			*LL*	*UL*	
Mother–adolescent communication (T1)	0.01	0.00	0.01	0.02	0.000 **
Participant younger	−0.16	0.09	−0.33	0.01	0.727
Mixed-gender dyad	0.03	0.09	−0.15	0.22	0.727
YoungerXMixed-gender dyad	0.14	0.13	−0.12	0.39	0.291
Sibling relationship quality (T1)	−0.05	0.02	−0.09	−0.00	0.052
Sibling relationship quality (T2)	0.17	0.02	0.13	0.21	0.000 **
Participant’s race—Black or African American (T1)	−0.03	0.13	−0.28	0.22	0.808
Participant’s race—Hispanic or Latino (T1)	−0.30	0.10	−0.49	−0.11	0.002 **
Participant’s race—other (T1)	−0.03	0.08	−0.19	0.12	0.667
Participant’s age (T1)	0.05	0.04	−0.04	0.13	0.301

*Note. N* = 1428, CI = confidence interval, LL = lower limit, UL = upper limit, ** *p* < 0.01, outcome = active coping strategies (T3).

**Table 21:** Path coefficients from predictors, interaction terms and covariates to the outcome.

Variables	*b*	*SE*	95% CI		*p*
			*LL*	*UL*	
Mother–adolescent communication (T1)	0.01	0.00	0.00	0.01	0.000 **
Participant younger	−0.14	0.07	−0.28	−0.01	0.033 *
Mixed-gender dyad	−0.03	0.07	−0.18	0.11	0.654
YoungerXMixed-gender dyad	0.08	0.10	−0.12	0.28	0.423
Sibling relationship quality (T1)	−0.06	0.02	−0.10	−0.02	0.002 **
Sibling relationship quality (T2)	0.11	0.02	0.08	0.15	0.000 **
Participant’s race—Black or African American (T1)	0.03	0.10	−0.17	0.23	0.744
Participant’s race—Hispanic or Latino (T1)	−0.14	0.07	−0.28	0.01	0.068
Participant’s race—other (T1)	−0.07	0.06	−0.19	0.05	0.261
Participant’s age (T1)	−0.00	0.03	−0.07	0.06	0.900

*Note. N* = 1428, CI = confidence interval, LL = lower limit, UL = upper limit, * *p* < 0.05, ** *p* < 0.01, outcome = distraction coping strategies (T3).

**Table 22:** Path coefficients from predictors, interaction terms and covariates to the outcome.

Variables	*b*	*SE*	95% CI		*p*
			*LL*	*UL*	
Mother–adolescent communication (T1)	0.01	0.00	0.01	0.02	0.000 **
Participant younger	−0.19	0.08	−0.36	−0.03	0.023 *
Mixed-gender dyad	−0.01	0.09	−0.19	0.17	0.915
YoungerXMixed-gender dyad	0.17	0.13	−0.08	0.42	0.188
Sibling relationship quality (T1)	−0.06	0.02	−0.10	−0.01	0.020 **
Sibling relationship quality (T2)	0.16	0.02	0.12	0.20	0.000 **
Participant’s race—Black or African American (T1)	−0.03	0.12	−0.27	0.21	0.821
Participant’s race—Hispanic or Latino (T1)	−0.28	0.09	−0.47	−0.10	0.003 **
Participant’s race—other (T1)	−0.03	0.08	−0.18	0.13	0.736
Participant’s age (T1)	0.04	0.04	−0.05	0.12	0.395

*Note. N* = 1428, CI = confidence interval, LL = lower limit, UL = upper limit, * *p* < 0.05, ** *p* < 0.01, outcome = problem-focused coping strategies (T3).

**Table 23:** Path coefficients from predictors, interaction terms and covariates to the outcome.

Variables	*b*	*SE*	95% CI		*p*
			*LL*	*UL*	
Father–adolescent communication (T1)	0.02	0.00	0.02	0.02	0.000 **
Participant younger	−0.18	0.08	−0.34	−0.02	0.031 *
Mixed-gender dyad	0.04	0.09	−0.13	0.22	0.625
YoungerXMixed-gender dyad	0.08	0.12	−0.15	0.32	0.485
Sibling relationship quality (T1)	−0.02	0.02	−0.07	0.02	0.320
Sibling relationship quality (T2)	0.14	0.02	0.10	0.18	0.000 **
Participant’s race—Black or African American (T1)	−0.00	0.12	−0.24	0.23	0.982
Participant’s race—Hispanic or Latino (T1)	−0.19	0.09	0.37	−0.01	0.039 *
Participant’s race—other (T1)	0.02	0.07	−0.06	0.10	0.668
Participant’s age (T1)	0.02	0.04	−0.06	0.10	0.668

*Note. N* = 1428, CI = confidence interval, LL = lower limit, UL = upper limit, * *p* < 0.05, ** *p* < 0.01, outcome = active coping strategies (T3).

**Table 24:** Path coefficients from predictors, interaction terms and covariates to the outcome.

Variables	*b*	*SE*	95% CI		*p*
			*LL*	*UL*	
Father–adolescent communication (T1)	0.01	0.00	0.01	0.02	0.000 **
Participant younger	−0.15	0.07	−0.28	0.02	0.027 *
Mixed-gender dyad	−0.03	0.07	−0.17	0.11	0.702
YoungerXMixed-gender dyad	0.04	0.10	−0.15	0.23	0.689
Sibling relationship quality (T1)	−0.04	0.02	−0.07	0.00	0.053
Sibling relationship quality (T2)	0.09	0.02	0.05	0.12	0.000 **
Participant’s race—Black or African American (T1)	0.05	0.10	−0.15	0.24	0.626
Participant’s race—Hispanic or Latino (T1)	−0.07	0.07	−0.21	0.08	0.366
Participant’s race—other (T1)	−0.04	0.06	−0.16	0.08	0.475
Participant’s age (T1)	−0.02	0.03	−0.09	0.04	0.521

*Note. N* = 1428, CI = confidence interval, LL = lower limit, UL = upper limit, * *p* < 0.05, ** *p* < 0.01, outcome = distraction coping strategies (T3).

**Table 25:** Path coefficients from predictors, interaction terms and covariates to the outcome.

Variables	*b*	*SE*	95% CI		*p*
			*LL*	*UL*	
Father–adolescent communication (T1)	0.02	0.00	0.02	0.02	0.000 **
Participant younger	−0.20	0.08	−0.36	−0.04	0.013 *
Mixed-gender dyad	0.00	0.09	−0.17	0.17	0.991
YoungerXMixed-gender dyad	0.10	0.12	−0.14	0.34	0.408
Sibling relationship quality (T1)	−0.03	0.02	−0.08	0.01	0.128
Sibling relationship quality (T2)	0.13	0.02	0.09	0.17	0.000 **
Participant’s race—Black or African American (T1)	0.00	0.12	−0.23	0.23	0.981
Participant’s race—Hispanic or Latino (T1)	−0.18	0.09	−0.36	−0.00	0.048 *
Participant’s race—other (T1)	0.03	0.07	−0.11	0.17	0.708
Participant’s age (T1)	0.01	0.04	−0.07	0.10	0.708

*Note. N* = 1428, CI = confidence interval, LL = lower limit, UL = upper limit, * *p* < 0.05, ** *p* < 0.01, outcome = problem-focused coping strategies (T3).

## Data Availability

The data presented in this study are available on request from the corresponding author due to participant anonymity.
